# Differential hypothalamic expression of Musashi protein 2 in Mongolian sheep with single and double ovulation

**DOI:** 10.3389/fvets.2026.1822626

**Published:** 2026-09-03

**Authors:** Jiaxin Fan, Penghui Li, Bingkun Teng, Haodong Liu, Qinqin Hao, Yang He, Mingyang Yu, Zelin Zhang, Xiaolong Li, Junzhe Yin, Wenchao Yang, Guifang Cao, Haijun Li, Xiaojuan Cao, Haiyan Ge, Yongqiang Li, Junguang Ren, Chenguang Du

**Affiliations:** 1 College of Veterinary Medicine, Inner Mongolia Agricultural University, Hohhot, China; 2 Key Laboratory of Veterinary Fundamentals and Disease Prevention and Control for Herbivorous Livestock in Inner Mongolia Autonomous Region, Hohhot, China; 3 Vocational and Technical College, Inner Mongolia Agricultural University, Baotou, China; 4 Inner Mongolia Huimu Animal Husbandry Co., Ltd., Xing’an League, China; 5 Inner Mongolia Grassland Black Bone Sheep Biotechnology Co., Ltd., Hohhot, China

**Keywords:** gonadotropin-releasing hormone, hypothalamic nucleus, Mongolian sheep, Musashi-2, reproductive rate

## Abstract

**Introduction:**

Musashi-2 (MSI2) is an RNA-binding protein involved in the regulation of mammalian reproduction. However, its expression characteristics and role in hypothalamic reproductive regulation in Mongolian sheep remain unclear. We investigated the differential expression of MSI2 in key hypothalamic nuclei of Mongolian ewes and analyzed its potential association with gonadotropin-releasing hormone (GnRH)-related neuroendocrine characteristics.

**Methods:**

Sixty 12-month-old Mongolian ewes were selected and divided into single corpus luteum (single-CL) and double corpora lutea (double-CL) groups. Immunofluorescence was used to detect the colocalization of MSI2 and GnRH in the supraoptic nucleus (SON), anteroventral periventricular nucleus (AVPV), arcuate nucleus (ARC), and paraventricular nucleus (PVN). Reverse transcription-quantitative PCR (RT-qPCR) and western blotting were performed to determine MSI2 mRNA and protein expression levels, as well as differences in GnRH protein expression. Sanger sequencing was used to preliminarily screen for MSI2 sequence variations, and enzyme-linked immunosorbent assay (ELISA) was performed to detect serum estradiol (E2), GnRH, follicle-stimulating hormone (FSH), luteinizing hormone (LH), and progesterone (P4) levels.

**Results:**

MSI2 was expressed in the SON, AVPV, ARC, and PVN of ewes in both groups. MSI2 mRNA and protein levels were significantly higher in the single-CL group. By contrast, GnRH protein expression was higher in the double-CL group, whereas MSI2 protein levels were significantly negatively correlated with GnRH protein levels in all examined hypothalamic nuclei. Immunofluorescence showed colocalization of MSI2 and GnRH, suggesting that MSI2 may be involved in GnRH-related neuroendocrine regulation. The double-CL group showed significantly higher serum E2, GnRH, LH, and P4 levels, but lower FSH levels. In the single-CL group, an A/G overlapping peak signal was observed at the c.66 site of exon 6 of MSI2, which was identified as a synonymous variant based on sequence alignment. In contrast, the corresponding site in the double-CL group showed a single A peak signal.

**Conclusion:**

Differential hypothalamic MSI2 expression may be associated with GnRH-related neuroendocrine differences between Mongolian ewes with single and double corpora lutea. This study provides a theoretical basis for further exploration of the potential role of MSI2 in ovulation-related traits in Mongolian sheep.

## 1 Introduction

Mongolian sheep are a representative meat sheep breed in the Inner Mongolia Autonomous Region. However, because they generally produce single lambs, their reproductive performance is relatively low (1). Previous studies have reported that Mongolian rams are usually first mated at 18 months of age, whereas Mongolian ewes are usually first mated at 8–12 months of age. Mongolian ewes exhibit seasonal estrus, mainly from September to November, with an estrous cycle of approximately 18.1 d and a gestation period of approximately 147.1 d. They usually lamb once per year, with an average lambing rate of approximately 103% and a lamb weaning survival rate of approximately 99% (2). Therefore, improving ovulation-related and prolificacy traits is important for enhancing the economic efficiency of Mongolian sheep production. The hypothalamic–pituitary–gonadal (HPG) axis regulates reproduction (3). Gonadotropin-releasing hormone (GnRH), synthesized and released by hypothalamic GnRH neurons, modulates the secretion of follicle-stimulating hormone (FSH) and luteinizing hormone (LH) through varying pulse frequencies and amplitudes, with higher frequencies favoring LH secretion and lower frequencies promoting FSH secretion (4). As downstream effector molecules, FSH and LH act on the gonads through the bloodstream, stimulating gonadal growth and gamete production while maintaining puberty onset and reproduction through the dynamic balance of sex steroid hormones (5).

Musashi-2 (MSI2), a conserved RNA-binding protein, regulates gene expression at the posttranscriptional level (6, 7). MSI2 is highly expressed in the mature hormone-producing cell lineages of the anterior pituitary, with MSI1 and MSI2 expression levels in the pituitary second only to those in the gonads (8, 9). GnRHR and FSHB mRNAs are direct targets of Musashi-dependent suppression during gonadotrope cell remodeling in the estrous cycle *in vivo* (8–12). Furthermore, high-throughput sequencing has revealed that MSI2 expression is associated with genes involved in steroid and aldosterone biosynthesis (13), suggesting that MSI2 may influence GnRH secretion by modulating the translation of pituitary-related mRNAs. However, direct evidence supporting the involvement of MSI2 in the regulation of gene expression in hypothalamic GnRH neurons remains limited.

Therefore, we aimed to determine the distribution of MSI2 in key hypothalamic nuclei in Mongolian sheep using immunofluorescence. Brain tissues from Mongolian sheep with double corpora lutea after ovulation were used as the experimental group, whereas tissues from ewes with a single corpus luteum served as the control group. Western blotting and reverse transcription quantitative PCR (RT-qPCR) analyses were employed to evaluate differences in MSI2 mRNA and protein expression in key hypothalamic nuclei between the single-CL and double-CL groups. Sanger sequencing was used to preliminarily screen for sequence variations in *MSI2*. Hormone level were assessed to clarify differences in hormone profiles between the single-CL and double-CL groups, with the aim of exploring potential correlations between reproductive hormones and ovulation-related traits. By examining the coexpression and changes in MSI2 and GnRH protein expression, we investigated the role of MSI2 in key hypothalamic nuclei in the single-CL and double-CL groups. The present study comprehensively analyzed the role of MSI2 as an important reproduction-related gene and provides a theoretical basis for further research on ovulation-related traits toward improving reproductive performance in Mongolian sheep.

## 2 Materials and methods

### 2.1 Ethics statement

The Mongolian sheep brain tissue used in this study was sourced from a breeding farm in Keyouqian Banner, Xingan League, northeastern Inner Mongolia, China. Euthanasia of the animals was approved by the Animal Welfare and Research Ethics Committee of Inner Mongolia Agricultural University (Approval No.:NND2025235).

### 2.2 Experimental materials

Sixty healthy, non-pregnant Mongolian ewes, with similar body condition, approximately 12 months of age, and an average body weight of 40.2 ± 3.5 kg, were selected for the experiment during September, the natural breeding season in Inner Mongolia. Estrus was detected four times daily for 30 min in each session using teaser rams and vaginal smears, and the onset of estrus was determined based on estrous behavior and vaginal smear results. Ewes identified as being in estrus were further examined via transrectal ultrasonography to monitor follicular development and ovulation. Bilateral ovarian laparoscopy was then performed to evaluate the presence and number of corpora lutea after ovulation. Depending on the number of corpora lutea in both ovaries after ovulation, the ewes were divided into single- and double-CL groups, with 30 ewes in each group. After slaughter, both ovaries were examined again to confirm the number of corpora lutea and the accuracy of grouping. The corpora lutea were light in color, soft in texture, and showed fresh bleeding points and clear ovulation depressions, suggestive of the early luteal stage after ovulation. The ovaries, oviducts, and uterus were examined grossly to exclude ewes with obvious reproductive tract abnormalities. The brain tissue and blood samples were aseptically collected from both groups and transported to the laboratory on dry ice. Fifteen brain tissue samples were randomly selected from each group and stored at −80 °C for subsequent molecular experiments, whereas the remaining brain tissues were fixed in 4% paraformaldehyde for immunofluorescence experiments. Serum samples were separated via centrifugation after the blood samples were allowed to stand at 25 °C for 20 min and centrifuged at 12,000 rpm for 25 min, and stored at −80 °C until further analysis. Representative gross ovarian morphology of single-CL and double-CL ewes is shown in Supplementary Figure 3.

### 2.3 Hypothalamic multiple immunofluorescence

The brain tissue from ewes with single and double corpora lutea was fixed in 4% paraformaldehyde (PFA, pH 7.4; Sigma-Aldrich, 158,127) for 10 days. The hypothalamic regions were isolated and sectioned into 50 μm-thick slices using a vibratome (Leica VT1000; Leica Microsystems, Wetzlar, Germany). All sections were washed three times in phosphate-buffered saline (PBS, 0.01 M, pH 7.4) for 5 min each time. The sections were permeabilized in pre-chilled methanol at −20 °C for 10 min, and rinsed three times in PBS. Subsequently, the brain sections were immersed in EDTA antigen retrieval solution (1×) and incubated in a water bath at 95 °C for approximately 25 min; thereafter, the sections were cooled to 20 °C and washed again in PBS. The sections were incubated overnight at 4 °C in a humidified chamber with the following primary antibodies: anti-MSI2 (1:200, rabbit; Ab76148; Abcam, Cambridge, United Kingdom) and anti-GnRH (1:200, rabbit; DF8553; Affinity). After three washes in PBS, a rabbit two-step detection kit (PV-9001) was used as the secondary antibody. Finally, fluorescent immunostaining was performed using a triple fluorescence immunohistochemistry kit (RS0036; Immunoway, San Jose, CA, United States) according to the manufacturer’s protocol. Laser confocal scanning parameters were set, and sections without primary antibodies were used as negative controls. The sections were mounted and imaged using a laser confocal microscope (Nikon C2; Nikon, Tokyo, Japan).

### 2.4 Protein extraction and Western blotting

The supraoptic nucleus (SON), anteroventral periventricular nucleus (AVPV), arcuate nucleus (ARC), and paraventricular nucleus (PVN) were dissected from the hypothalamus of Mongolian ewes with single and double corpora lutea. Each sample was homogenized in high-efficiency RIPA lysis buffer containing protease inhibitors using an electric homogenizer. Samples were sonicated (15 s/time, 90 V, three times) (11), and the homogenate was centrifuged at 12,000 × *g* for 10 min at 4 °C. Protein concentrations were determined using the BCA method (Pierce Protein Assay Kit, Thermo Fisher Scientific, Waltham, MA, United States) and adjusted to 0.7 μg/μL before adding loading buffer. Then, 100 μL of protein sample was mixed with 25 μL of loading buffer. After denaturation at 95 °C for 5 min, the samples were subjected to polyacrylamide gel electrophoresis (12% separating gel and 5% stacking gel), with 20 μL of the final mixture, corresponding to 11.2 μg of total protein, was loaded per well. Proteins were transferred onto polyvinylidene difluoride membrane. The membrane was blocked with 5% non-fat milk in 1 × TBST buffer at room temperature for 1 h. and then incubated overnight at 4 °C with the following primary antibodies: anti-MSI2 (1:1,000, rabbit; Ab76148; Abcam), anti-GnRH (1:2,000, rabbit; DF8553; Affinity), and anti-*β*-Tubulin (1:2,500, rabbit; Ab215037, Abcam). The next day, the membrane was washed three times with 1 × TBS buffer, incubated with secondary antibody (goat anti-rabbit IgG, 1:10,000; LI-COR Biosciences, Lincoln, NE, United States) at 37 °C on a shaker for 1 h, and washed again with 1 × TBS. The membrane was then scanned and imaged using a protein imaging system (Li-Cor, Odyssey-CLX, United States). Fluorescence values of target and reference proteins were statistically analyzed.

### 2.5 Quantitative reverse transcription polymerase chain reaction

The brain tissue samples from Mongolian sheep with single and double corpora lutea that had been stored at −80 °C were retrieved, and the hypothalamic nuclei, including the SON, AVPV, ARC, and PVN, were carefully dissected. Total RNA was extracted from each nucleus using the MagicPure Total RNA Kit (EC521) according to the manufacturer’s instructions. Complementary DNA (cDNA) was synthesized from total RNA using the Takara PrimeScript RT Master Mix (Perfect Real Time, RR036A) cDNA reverse transcription kit. Quantitative PCR was performed on a CFX96 instrument (Bio-Rad Laboratories, Hercules, CA, United States). Primer sequences were as follows: MSI2 (forward: 5′-TTAGACTCCAAGACGATTGAC-3′; reverse: 5′-GCGTCCATGGTGTAAGGCAGT-3′); *β*-actin (forward: 5′-TTTCTACTTTGCCAGCAA-3′; reverse: 5′-CGAAGGAAGTCCAATGTC-3′). The PCR program included a denaturation step (95 °C for 30 s), followed by 40 cycles of amplification and quantification (95 °C for 30 s, 56 °C/60 °C for 30 s, and 72 °C for 30 s). Data were analyzed using the 2^−ΔΔCT^ method and were, normalized to *β*-actin expression.

### 2.6 Sanger sequencing

#### 2.6.1 DNA extraction

The brain tissue samples from Mongolian sheep with single and double corpora lutea that had been stored at −80 °C were retrieved. The hypothalamic tissue was dissected from both groups.

Genomic DNA was extracted from three hypothalamic tissue samples per group (*n* = 3 single-CL and *n* = 3 double-CL samples) using the EasyPure Genomic DNA Kit (EE101; TransGen Biotech, China) according to the manufacturer’s instructions. The purified DNA samples were then subjected to Sanger sequencing, which was performed by Sangon Biotech Co., Ltd. (Shanghai, China).

#### 2.6.2 Primer design and synthesis

The primers were designed based on approximately 300 bp sequences upstream and downstream of the target gene locus; they were designed and synthesized by Sangon Biotech (Shanghai, China). The primer sequences used are listed in Table 1.

**Table 1:** Primers used in the present study.

Gene names	Primer sequences (5′–3′)	Size (bp)
1/2/3	F: CGTCAGCGGCATTGGTTAC R: CCTGAGCCCCGTTCTAAATC	1,212 bp
4	F: CCCTTGGCTCTGCGTTCT R: GAGAGGGCGCTGTCTCTTTAAAC	272 bp
5	F: TTTGAGAAGTTACTGAAAGCTGGGT R: GGAATGGCTGACTAGCAGACAC	366 bp
6	F: TTTTGCAACCTCATGGACTGTAG R: GAAACTGACCCCAGGTATTAGG	373 bp
7	F: GGCTTGTTCAACAGCTCAGTG R: AGCATCTTCCCTGGGTTTCCT	629 bp
8	F: CAAATAGGTTTTAGTGAGCAGCATG R: TAACGGTCCGGTATCAACTCTAGG	363 bp
9	F: GTAAATTTCCTGTTTGCTTCCG R: CCCTACCAACTTAACCATTGTCTC	513 bp
10	F: CATTTACTCGGCGTGGCT R: TGGGTCAGGCATCTGTATGTTTC	415 bp
11	F: ATAGGGTCTCATCACCAGCAGG R: CCGGCACCCATGAAACACT	471 bp
12	F: CTTCAGCACTTTCCGCCAG R: AGCCCACTAAATGCTAAGACTATCG	315 bp
13	F: GACAGACGGGAGGGAATCAG R: GCGTCCGAAGTCCACAAGAA	501 bp
14	F: GAGTCCCTGCTGATGTCTTCG R: TGCCTTAGCCTGTTTCCTTTA	699 bp
15	F: CAGGGCTTGGGAGGATGT R: GGATGAGAGATTAAAAGGCTGG	418 bp

#### 2.6.3 PCR amplification

PCR amplification was performed using genomic DNA from Mongolian sheep as a template. The PCR mixture consisted of 1 μL template DNA, 1 μL each of forward and reverse primers, 1 μL dNTP mix, 2.2 μL Taq Buffer (with MgCl_2_), 0.2 μL Taq DNA polymerase, and dd H_2_O to a final volume of 25 μL. The thermal cycling conditions were as follows: pre-denaturation at 95 °C for 5 min; 30 cycles of denaturation at 94 °C for 30 s, annealing at 63 °C for 30 s, and extension at 72 °C for 30 s; followed by a final extension at 72 °C for 10 min and storage at 4 °C. The PCR products were detected via electrophoresis on a 1% agarose gel.

### 2.7 Hormone assay

Serum samples from Mongolian ewes in the single and double-CL groups stored at −80 °C were retrieved. Levels of reproductive hormones, namely estradiol (E2), gonadotropin-releasing hormone (GnRH), progesterone (P4), follicle-stimulating hormone (FSH), and luteinizing hormone (LH), were measured using enzyme-linked immunosorbent assay (ELISA). All ELISA kits were purchased from FineTest (Wuhan Fine Biotech Co., Ltd., Wuhan, China), and all procedures were performed according to the manufacturer’s instructions. The catalog numbers and sensitivities were as follows: E2, EU0390, 7.5 pg./mL; GnRH, ESH0062, 18.75 pg./mL; P4, ESH0086, 0.188 ng/mL; FSH, ESH0028, 1.875 mIU/mL; and LH, ESH0034, 0.563 mIU/mL. Absorbance was measured at 450 nm.

### 2.8 Statistical analysis

Experimental data were subjected to *t*-tests, Pearson correlation analysis, 95% confidence intervals, and two-tailed *p*-values using GraphPad Prism 8.0 software. Relative quantification was performed using the 2^−ΔΔCt^ method, with *β*-actin used as an internal reference gene to normalize target gene expression levels. Differences between the two groups were analyzed using an unpaired two-tailed Student’s *t*-test. RT-qPCR and western blot analyses were performed using four biological replicates per group. Data are presented as the mean ± standard deviation. A value of *p* < 0.05 was considered statistically significant.

## 3 Results

### 3.1 Coexpression analysis of MSI2 and GnRH in key hypothalamic nuclei of Mongolian sheep in the single-CL and double-CL groups

**Figure 1:**
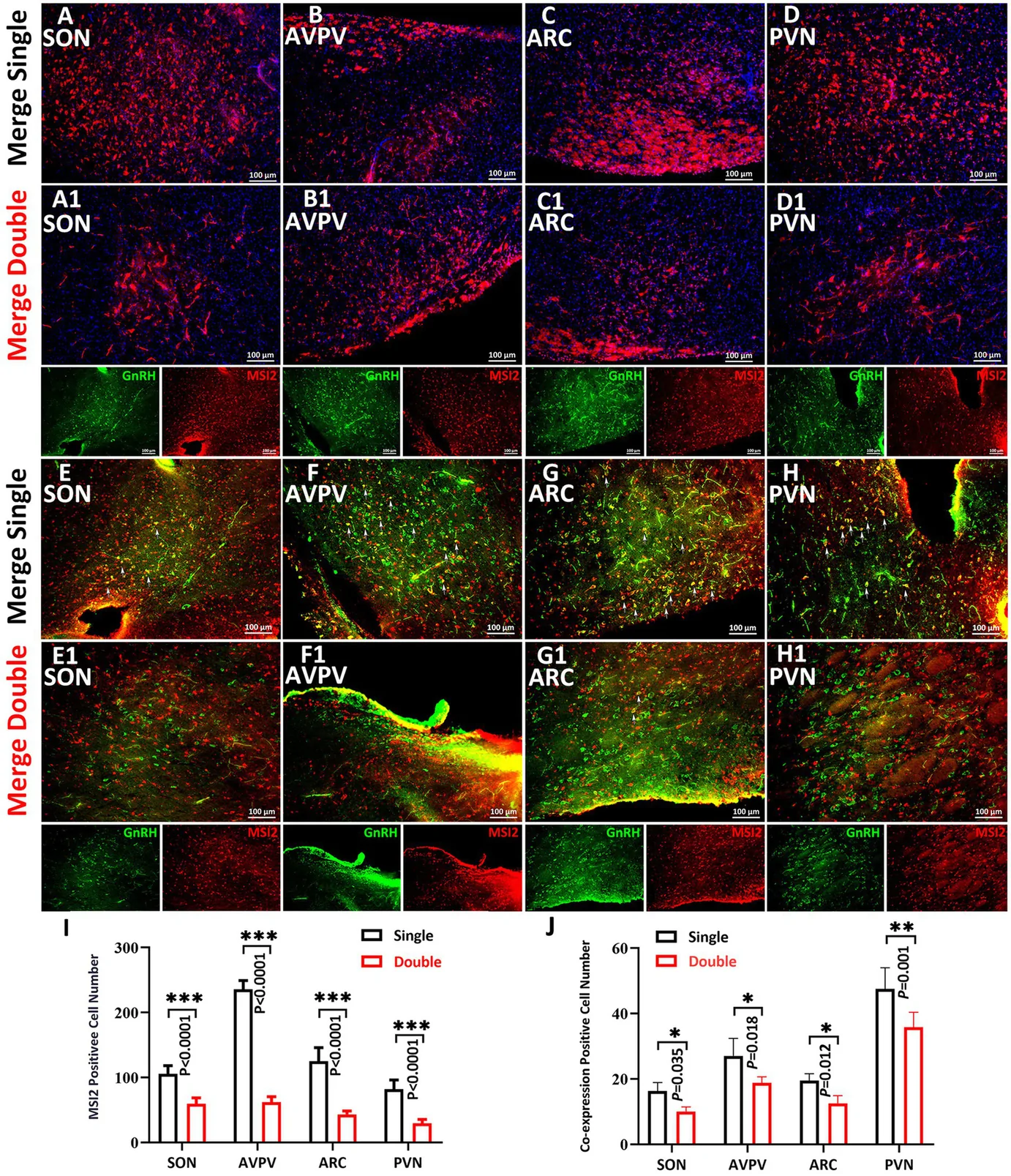
Immunofluorescence analysis of MSI2 and GnRH expression in hypothalamic nuclei of Mongolian sheep. MSI2, Musashi-2 protein (red); GnRH, gonadotropin-releasing hormone protein (green); Single, hypothalamus of Mongolian sheep with a single corpus luteum; Double, hypothalamus of Mongolian sheep with double corpora lutea; SON, supraoptic nucleus; AVPV, anteroventral periventricular nucleus; ARC, arcuate nucleus; PVN, paraventricular nucleus. **(A)** MSI2 expression in the SON of the hypothalamus from sheep with a single corpus luteum. **(A1)** MSI2 expression in the SON of the hypothalamus from sheep with double corpora lutea. **(B)** MSI2 expression in the AVPV of the hypothalamus from sheep with a single corpus luteum. **(B1)** Immunofluorescence analysis of MSI2 expression in the AVPV of the hypothalamus from sheep with double corpora lutea. **(C)** MSI2 expression in the ARC of the hypothalamus from sheep with a single corpus luteum. **(C1)** MSI2 expression in the ARC of the hypothalamus from sheep with double corpora lutea. **(D)** Immunofluorescence analysis of MSI2 expression in the PVN of the hypothalamus from sheep with a single corpus luteum. **(D1)** Immunofluorescence analysis of MSI2 expression in the PVN of the hypothalamus from sheep with double corpora lutea. **(E)** Coexpression of MSI2 and GnRH in the SON of the hypothalamus from sheep with a single corpus luteum. **(F)** Coexpression of MSI2 and GnRH in the AVPV of the hypothalamus from sheep with a single corpus luteum. **(G)** Immunofluorescence analysis of MSI2 and GnRH coexpression in the ARC of the hypothalamus from sheep with a single corpus luteum. **(H)** Coexpression of MSI2 and GnRH in the PVN of the hypothalamus from sheep with a single corpus luteum. **(E1)** Immunofluorescence analysis of MSI2 and GnRH coexpression in the SON of the hypothalamus from sheep with double corpora lutea. **(F1)** Coexpression of MSI2 and GnRH in the AVPV of the hypothalamus from sheep with double corpora lutea. **(G1)** Immunofluorescence analysis of MSI2 and GnRH coexpression in the ARC of the hypothalamus from sheep with double corpora lutea. **(H1)** Coexpression of MSI2 and GnRH in the PVN of the hypothalamus from sheep with double corpora lutea. **(I)** Quantification of MSI2-positive neurons in the SON, AVPV, ARC, and PVN of sheep with single and double corpora lutea. **(J)** Quantification of neurons coexpressing MSI2 and GnRH in the SON, AVPV, ARC, and PVN of sheep with single and double corpora lutea.

Immunofluorescence analysis (Figures 1A–D) demonstrated the specific expression of MSI2 in key hypothalamic nuclei (SON, AVPV, ARC, and PVN) of Mongolian sheep in the single-CL and double-CL groups. Compared with the double-CL group, the number of MSI2-expressing neurons in the SON, AVPV, ARC, and PVN was significantly higher in the single-CL group (Figure 1I) (*P* < 0.001). We also used dual-label immunofluorescence staining to examine the coexpression of GnRH and MSI2 in key hypothalamic nuclei of Mongolian ewes in the single-CL and double-CL groups. Notably, we found (Figures 1E–H) that MSI2 and GnRH were coexpressed in the SON, AVPV, ARC, and PVN, with a significantly higher coexpression level in the single-CL than in the double-CL group (Figure 1J) (*P* < 0.05). These results suggest that changes in MSI2 expression may be associated with GnRH-related neuroendocrine characteristics.

### 3.2 Analysis of MSI2 mRNA expression in key hypothalamic nuclei of Mongolian sheep in the single-CL and double-CL groups

**Figure 2:**
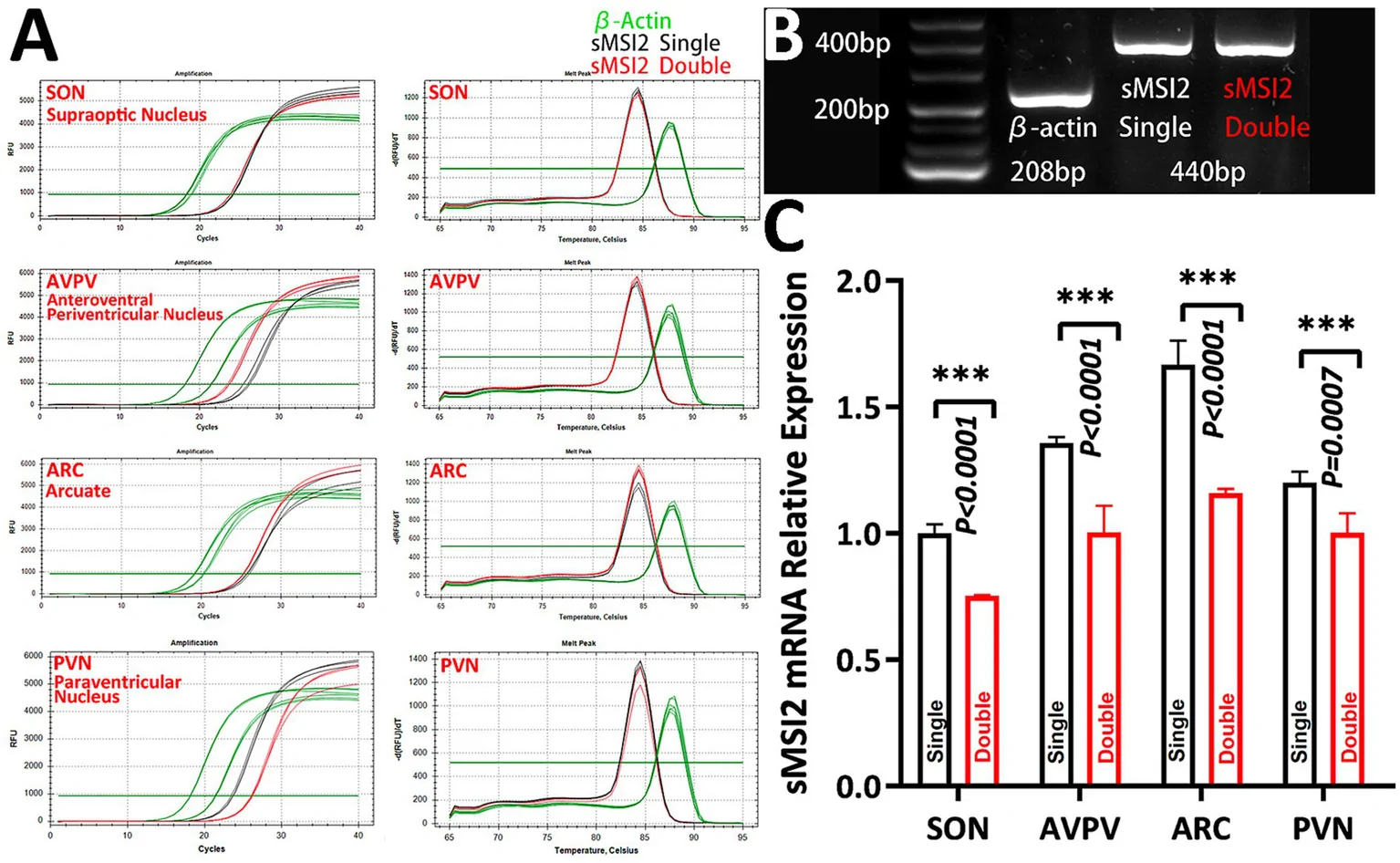
Differential MSI2 mRNA Expression in Hypothalamic Nuclei of Mongolian Sheep with Single and Double Corpora Lutea.*Indicates a significant difference (*p* < 0.05); **indicates a highly significant difference (*p* < 0.01); ***indicates an extremely significant difference (*p* < 0.001). MSI2, Musashi-2; *β*-actin, beta-actin; Single, brain tissue from sheep with a single corpus luteum; Double, brain tissue from sheep with double corpora lutea; SON, supraoptic nucleus; AVPV, anteroventral periventricular nucleus; ARC, arcuate nucleus; PVN, paraventricular nucleus. **(A)** qPCR amplification and melting curves of MSI2 in the SON, AVPV, ARC, and PVN of the single-CL and double-CL groups. **(B)** Agarose gel electrophoresis image of PCR products. **(C)** Differential MSI2 mRNA expression in the SON, AVPV, ARC, and PVN between sheep with single and double corpora lutea.

We conducted RT-qPCR to detect relative *MSI2* mRNA expression levels in key hypothalamic nuclei of Mongolian sheep in the single-CL and double-CL groups. Accordingly, we detected significant differences in *MSI2* mRNA expression levels in the SON, AVPV, ARC, and PVN between the single-CL and double-CL groups (*p* < 0.05). Specifically, *MSI2* mRNA expression levels in the SON (*p* < 0.0001), AVPV (*p* < 0.0001), ARC (*p* < 0.0001), and PVN (*p* = 0.0007)were significantly higher in the single-CL group than in the double-CL group (Figure 2C). Analysis of the RT-qPCR results confirmed the specificity of the MSI2 primers, with the amplicons showing the expected sizes, thereby validating the reliability of the experimental results (Figures 2A,B).

### 3.3 Analysis of MSI2 and GnRH protein expression in key hypothalamic nuclei of Mongolian sheep in the single-CL and double-CL groups

**Figure 3:**
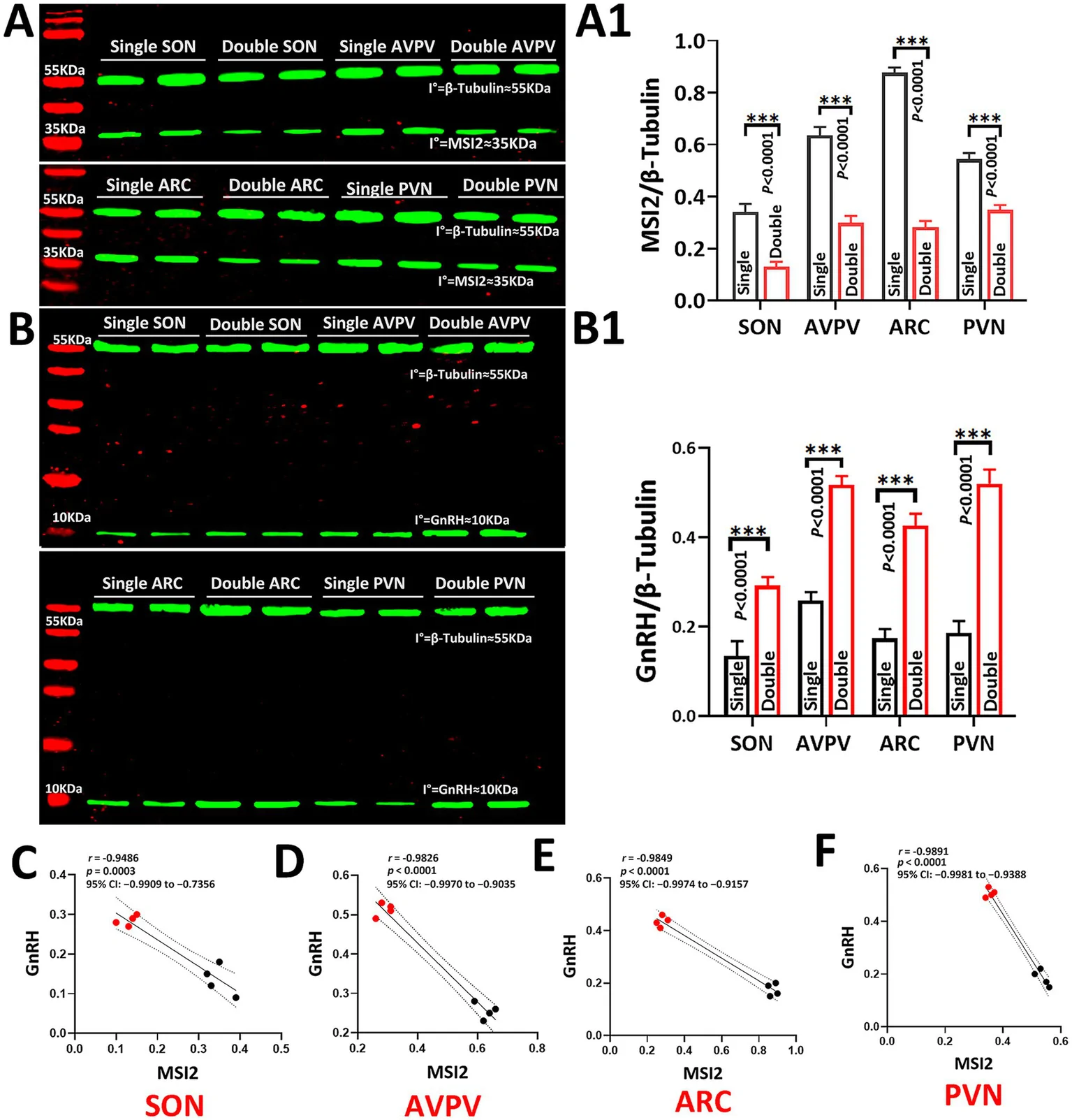
Differential expression and correlation analysis of MSI2 and GnRH proteins in hypothalamic nuclei of Mongolian sheep with single and double corpora lutea.*Indicates a significant difference (*p* < 0.05); **indicates a highly significant difference (*p* < 0.01); ***indicates an extremely significant difference (*p* < 0.001). MSI2, Musashi-2; β-tubulin, reference protein; Single, brain tissue from Mongolian sheep with a single corpus luteum; Double, brain tissue from Mongolian sheep with double corpora lutea; SON, supraoptic nucleus; AVPV, anteroventral periventricular nucleus; ARC, arcuate nucleus; PVN, paraventricular nucleus; Black, single corpus luteum; Red, double corpora lutea; r, Pearson correlation coefficient. **(A)** Changes in MSI2 protein expression in the SON, AVPV, ARC, and PVN of Mongolian sheep with single and double corpora lutea. **(A1)** Histogram showing changes in MSI2 protein expression in the SON, AVPV, ARC, and PVN of Mongolian sheep with single and double corpora lutea. **(B)** Changes in GnRH protein expression in the SON, AVPV, ARC, and PVN of Mongolian sheep with single and double corpora lutea. **(B1)** Histogram showing changes in GnRH protein expression in the SON, AVPV, ARC, and PVN of Mongolian sheep with single and double corpora lutea. **(C)** Correlation analysis between MSI2 and GnRH expression in the SON. **(D)** Correlation analysis between MSI2 and GnRH expression in the AVPV. **(E)** Correlation analysis between MSI2 and GnRH expression in the ARC. **(F)** Correlation analysis between MSI2 and GnRH expression in the PVN.

We performed western blotting to detect MSI2 protein expression levels in key hypothalamic nuclei of Mongolian ewes in the single-CL and double-CL groups. We observed significant differences in MSI2 protein expression in the SON, AVPV, ARC, and PVN between the two groups. Compared with the double-CL group, MSI2 protein expression levels in the SON, AVPV, ARC, and PVN were significantly higher in the single-CL group (all *p* < 0.0001) (Figures 3A,A1). These findings were consistent with the mRNA expression pattern, further confirming the high MSI2 expression in key hypothalamic nuclei of the single-CL group.

To further explore the potential relationship between MSI2 and GnRH, we analyzed GnRH protein expression in key hypothalamic nuclei of Mongolian ewes in the single-CL and double-CL groups. We detected GnRH protein expression in the SON, AVPV, ARC, and PVN of both groups. Compared with the single-CL group, GnRH protein expression levels in the SON, AVPV, ARC, and PVN were significantly higher in the double-CL group (all *p* < 0.0001) (Figures 3B,B1). Furthermore, we conducted Pearson correlation analysis to evaluate the relationship between MSI2 and GnRH protein expression levels. MSI2 and GnRH protein expression levels were significantly negatively correlated in the SON, AVPV, ARC, and PVN, with Pearson correlation coefficients of −0.9486, −0.9826, −0.9849, and −0.9891, respectively. The corresponding *p*-values and 95% confidence intervals (CIs) were as follows: SON, *p* = 0.0003, 95% CI: −0.9909 to −0.7356; AVPV, *p* < 0.0001, 95% CI: −0.9970 to −0.9035; ARC, *p* < 0.0001, 95% CI: −0.9974 to −0.9157; and PVN, *p* < 0.0001, 95% CI: −0.9981 to −0.9388 (Figures 3C–F).

These results suggest that lower MSI2 expression in key hypothalamic nuclei of the double-CL group may be associated with higher GnRH protein expression and may contribute to GnRH-related neuroendocrine differences and ovulation-related traits. However, whether MSI2 directly regulates GnRH expression and the underlying mechanism remain to be validated through functional experiments. Biological replicate verification results of all western blot experiments are presented in Supplementary Figure 2.

### 3.4 MSI2 gene mutation analysis

**Figure 4:**
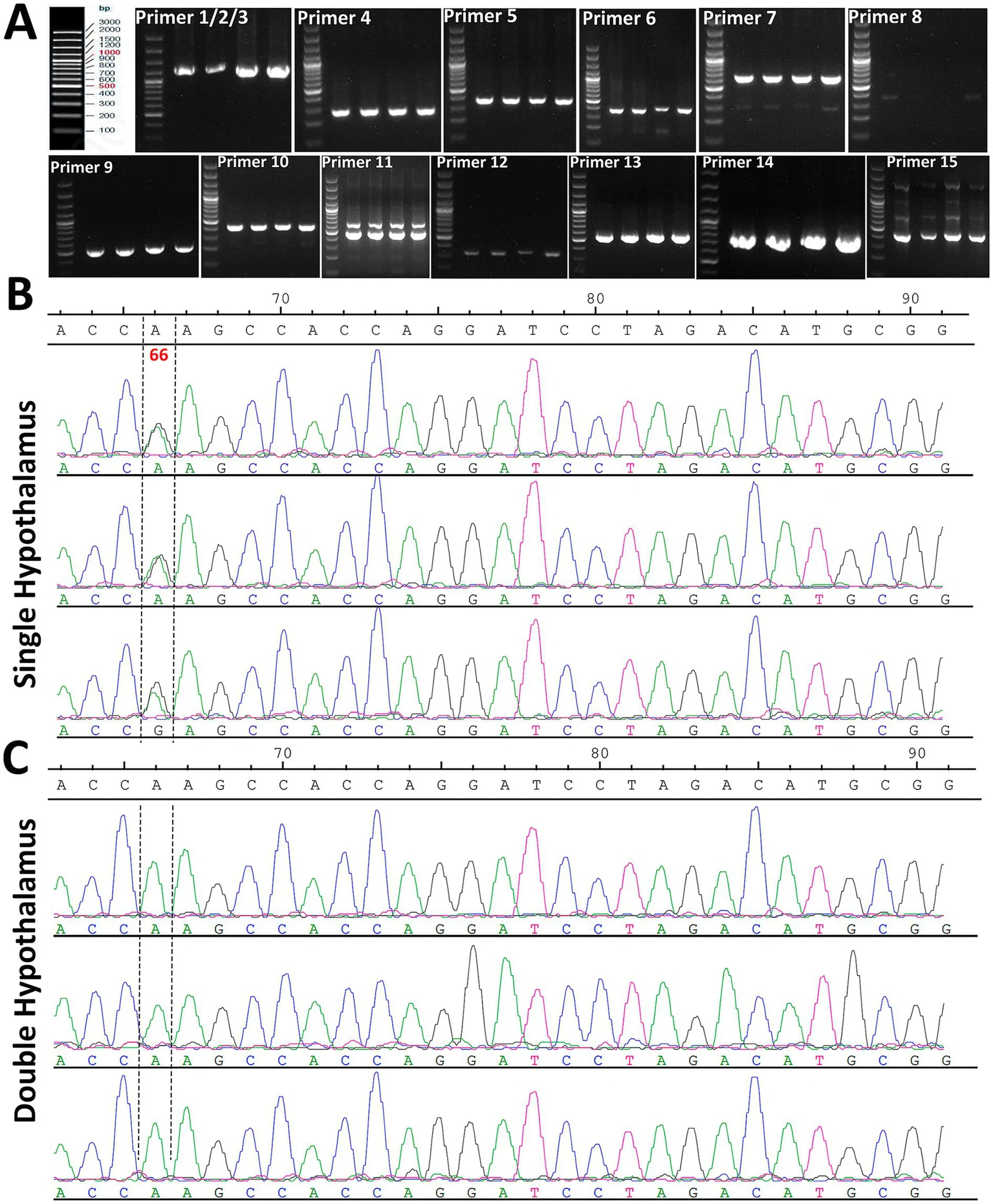
Sequencing Results of the *MSI2* Locus in Mongolian Sheep Single, hypothalamus of Mongolian sheep with a single corpus luteum; Double, hypothalamus of Mongolian sheep with double corpora lutea; 66, position 66 in exon 6. **(A)** 1% agarose gel electrophoresis showing PCR products amplified using primer pairs targeting *MSI2* exons/regions 1–15. M, 100–3,000 bp DNA ladder (Ladder-K); Primer 1–3 and Primer 4–15, amplification bands generated by each primer pair. **(B)** In the hypothalamus of sheep with a single corpus luteum, MSI2 at position 66 in exon 6 exhibited overlapping A and G peaks, expressed as A/G. **(C)** In the hypothalamus of sheep with double corpora lutea, MSI2 at position 66 in exon 6 exhibited a single A peak, expressed as A/A.

All primer pairs targeting *MSI2* exons/regions 1–15 yielded sharp, specific bands of the expected sizes in 1% agarose gel electrophoresis (Figure 4A). In the present study, we compared MSI2 sequences in the hypothalamus of Mongolian sheep from the single-CL and double-CL groups (*n* = 3 per group) to detect potential sequence variations. Sanger sequencing revealed an overlapping peak, indicating a heterozygous A/G substitution at position c.66 of exon 6 of the *MSI2* gene in the single-CL group (SNP g.7828531). Sequence alignment identified this as a synonymous variant with no change in the encoded proline residue (Figure 4B). In contrast, the corresponding site in the double-CL group showed only a single A peak, consistent with homozygosity for the reference allele (A/A) (Figure 4C).

### 3.5 Serum hormone level analysis

**Figure 5:**
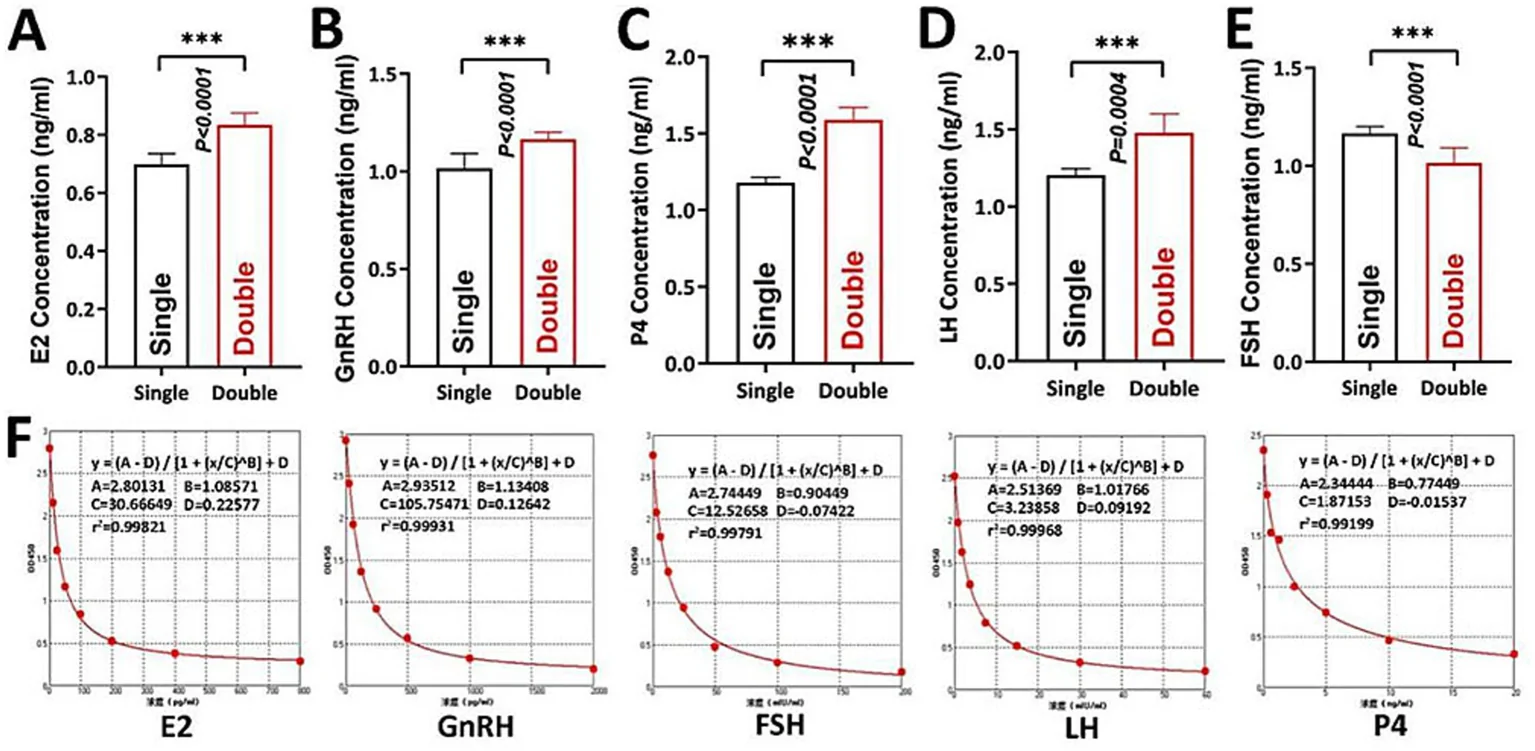
Differential Hormone Expression in Mongolian Sheep with Single and Double Corpora Lutea.*Indicates a significant difference (*p* < 0.05); **indicates a highly significant difference (*p* < 0.01); ***indicates an extremely significant difference (*p* < 0.001). Single, Mongolian sheep with a single corpus luteum; Double, Mongolian sheep with double corpora lutea; E2, estradiol; GnRH, gonadotropin-releasing hormone; FSH, follicle-stimulating hormone; LH, luteinizing hormone; P4, progesterone. **(A–E)** Serum concentrations of E2, GnRH, P4, LH, and FSH in Mongolian sheep with single corpus luteum (black) and double corpora lutea (red). **(F)** Linear regression analysis of E2, GnRH, FSH, LH, and P4 concentrations and absorbance values.

For hormone level analysis, serum samples from Mongolian ewes in the single-CL group served as the control group, whereas those from the double-CL group served as the experimental group, and serum hormone levels were compared between groups. Compared with the single-CL group, serum estradiol (E2; *p* < 0.001), GnRH (*p* < 0.001), LH (*p* < 0.001), and progesterone (P4; *p* < 0.001) levels were significantly higher in the double-CL group (Figures 5A–D). In contrast, the FSH level (*p* = 0.0023) was significantly higher in the single-CL than in the double-CL group (Figure 5E). Standard curve analysis showed good fitting performance for E2, GnRH, LH, P4, and FSH, with r^2^ values >0.95 (Figure 5F), indicating the reliability of the assay results.

Furthermore, serum GnRH levels were significantly higher in the double-CL than in the single-CL group (Figure 5B), consistent with the increased hypothalamic GnRH protein expression observed in the double-CL group. These findings suggest that reduced MSI2 expression may be associated with GnRH-related neuroendocrine differences and alterations in reproductive hormone profiles. However, the underlying regulatory mechanism requires further functional validation.

## 4 Discussion

The HPG axis is a key neuroendocrine center that regulates reproductive function in mammals. Key hypothalamic nuclei play important roles in coordinating reproductive activities. Kisspeptin neurons in the ARC and AVPV influence the function of the HPG axis (14). Kisspeptin neurons in the ARC act as a GnRH pulse generator, stimulating GnRH secretion through interactions with coexpressed Kiss-1R receptors on GnRH neurons (14–16). In contrast, kisspeptin neurons in the AVPV regulate the preovulatory GnRH and LH surge, as well as ovulation (17, 18). Additionally, corticotropin-releasing hormone (CRH) neurons in the PVN modulate reproductive function through the regulation of LH pulse frequency (19–21). The SON acts as a critical site for oxytocin synthesis and release, thereby controlling parturition and lactation (22). The present study investigated the differential expression of MSI2 in key hypothalamic nuclei of Mongolian ewes with single and double corpora lutea and analyzed its potential association with GnRH-related neuroendocrine activity and ovulation-related traits.

The present findings provide a theoretical basis for further exploration of the molecular mechanisms underlying reproductive function and development in sheep. Immunofluorescence analyses confirmed the specific hypothalamic expression of MSI2 in the SON, AVPV, ARC, and PVN of Mongolian sheep, with significantly higher MSI2 mRNA and protein expression levels in the single-CL than in the double-CL group. The findings further revealed that MSI2 colocalized with GnRH in these hypothalamic nuclei and exhibited a significant negative correlation with GnRH expression levels.

Previous studies have reported that MSI2 is expressed in ovarian granulosa cells of Mongolian sheep, with higher expression levels in single dominant follicles than in double dominant follicles. In addition, MSI2 expression was associated with genes related to follicular development and steroidogenesis, including CYP17A1, ESR2, StAR, and HSD3B1 (1). These findings are consistent with the overall trend observed in the present study, in which MSI2 expression in key hypothalamic nuclei was lower in the double-CL than in the single-CL group, suggesting that reduced MSI2 expression may be associated with double-CL status and ovulation-related traits in Mongolian sheep. In addition, previous studies have shown that knocking out *Msi2* in the dentate gyrus and ovary of female mice increased follicle number and litter size and altered gonadotropin secretion and the expression of genes related to ovarian steroidogenesis (23). These findings suggest that MSI2 may participate in reproductive regulation at both the neuroendocrine and ovarian levels. Combined with the colocalization of MSI2 and GnRH in key hypothalamic nuclei and the significant negative correlation between their protein expression levels observed in the present study, these results suggest that changes in MSI2 expression may be associated with GnRH-related neuroendocrine differences and ovulation-related traits. However, the specific underlying mechanism requires further validation.

Sanger sequencing results suggested possible MSI2 sequence differences between the single-CL and double-CL groups. In samples from the single-CL group, an A/G overlapping peak was identified at position c.66 of exon 6 of *MSI2*, suggesting a possible heterozygous A/G variant at this site. In contrast, the corresponding site in the double-CL group showed only a single A peak, consistent with homozygosity for the reference allele (A/A). Sequence alignment showed that this variant was synonymous and did not alter the encoded amino acid sequence. However, whether this variant affects MSI2 expression or function remains unclear. Therefore, future studies should expand the sample size further and incorporate functional validation experiments to clarify the potential biological significance of this synonymous variant and its relationship with MSI2 expression and ovulation-related traits.

Serum analyses revealed significantly higher levels of E2, GnRH, LH, and P4 and significantly lower FSH levels in double-CL than in single-CL Mongolian sheep. The elevated GnRH and LH levels in double-CL sheep likely reflect more frequent pulsatile GnRH secretion. The frequency and amplitude of pulsatile GnRH secretion are critical regulators of FSH and LH secretion, with high-frequency GnRH pulses preferentially stimulating LH secretion, whereas low-frequency pulses promote FSH secretion. The elevated GnRH and LH levels in double-CL sheep may therefore reflect differences in GnRH-related neuroendocrine activity. Additionally, increased E2 and P4 levels may regulate FSH synthesis and secretion through the hypothalamic–pituitary axis, thereby indirectly supporting follicle development and maintenance (24). Furthermore, the lower FSH levels in double-CL Mongolian sheep may be associated with negative feedback from high concentrations of estrogen and inhibin. Inhibin, acting synergistically with follistatin, suppresses activin activity and thereby inhibits FSH secretion (24). These hormonal differences suggest that the single-CL and double-CL groups exhibit distinct reproductive endocrine profiles. We hypothesized that MSI2 indirectly modulates the dynamic balance among FSH, LH, E2, and P4 by regulating GnRH secretion, thereby influencing follicle development and ovulation. This proposed mechanism is consistent with findings reported by Moreira et al., who demonstrated that MSI2 influences reproductive hormone secretion patterns by regulating the translation of gonadotropin target mRNAs during the estrous cycle in mice (10). However, direct experimental evidence supporting MSI2-mediated regulation of GnRH neurons remains lacking. Future studies should further investigate the direct and indirect regulatory mechanisms of MSI2 on GnRH neurons to elucidate fully its role in reproductive traits in Mongolian sheep.

## 5 Conclusion

MSI2 was expressed in the SON, AVPV, ARC, and PVN of Mongolian ewes, and its mRNA and protein expression levels were higher in the single-CL than in the double-CL group. In contrast, GnRH protein expression was higher in the double-CL group, whereas MSI2 protein levels were negatively correlated with GnRH protein levels in these hypothalamic nuclei. Serum hormone analysis showed that, compared with the single-CL, the double-CL group had higher levels of E2, GnRH, LH, and P4 but lower FSH levels, suggesting distinct reproductive endocrine characteristics between the two groups. Sanger sequencing preliminarily suggested possible *MSI2* sequence differences between ewes in the single-CL and double-CL groups. Overall, hypothalamic MSI2 expression may be associated with GnRH-related neuroendocrine differences and ovulation-related traits in Mongolian ewes. Future studies should expand the sample size further and incorporate functional validation and dynamic endocrine monitoring to clarify the potential role of MSI2 in GnRH-related neuroendocrine regulation and ovulation-related traits in sheep.

## Data Availability

All raw sequencing data generated in this study have been deposited in the Figshare repository and are publicly available under the DOI: https://doi.org/10.6084/m9.figshare.32725485.
