# Supplementary material for: Differential hypothalamic expression of Musashi protein 2 in Mongolian sheep with single and double ovulation
Source: Front Vet Sci. 2026 Sep 3;13:1822626. doi: 10.3389/fvets.2026.1822626 (PMC13595138; doi:10.3389/fvets.2026.1822626)

Supplementary Figure 2

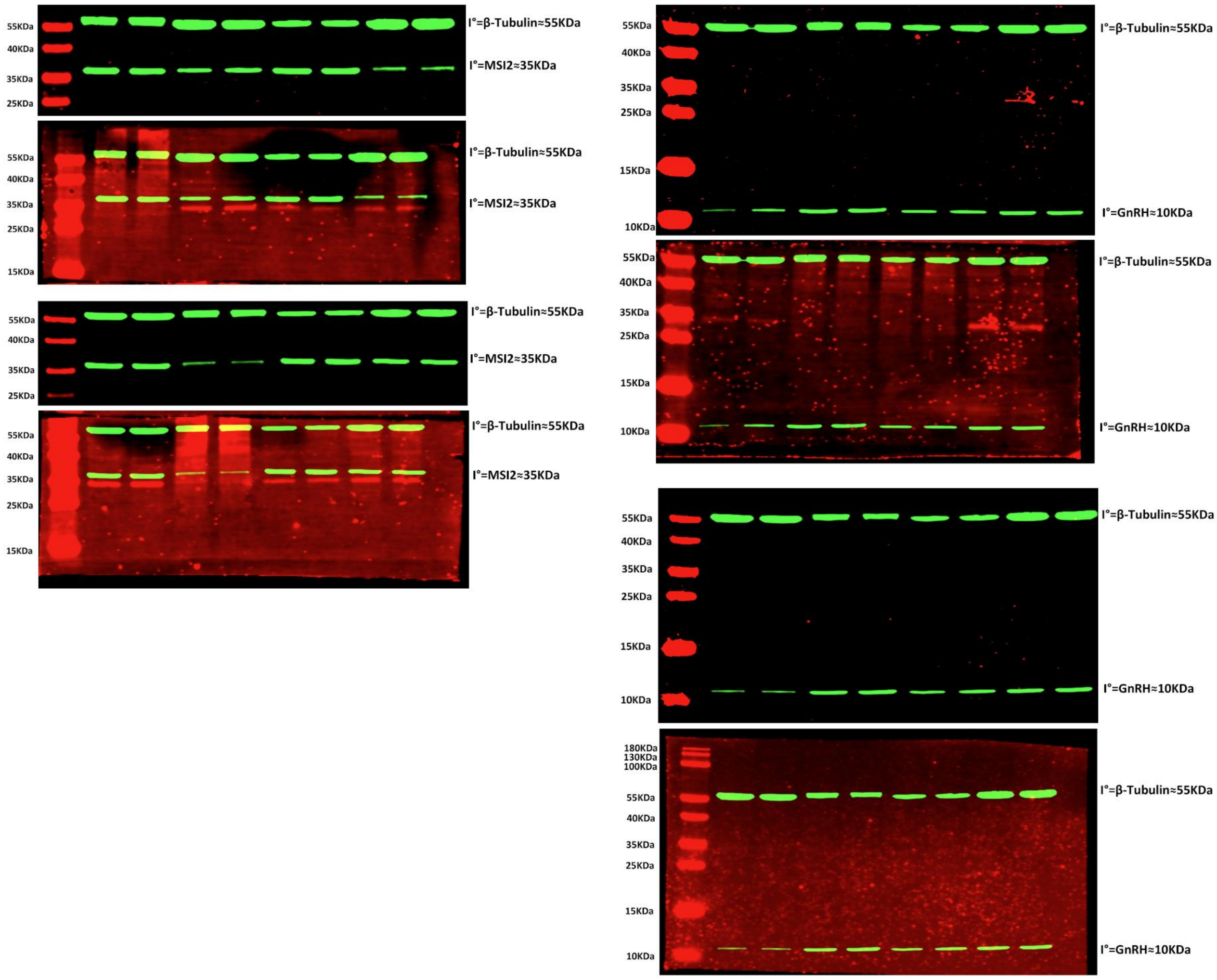

Supplement: Supplementary file 1 [file Image_2.PDF]
